# Environmental insights from non-target detections in urban eDNA metabarcoding

**DOI:** 10.1016/j.isci.2026.114632

**Published:** 2026-01-07

**Authors:** Yujin Kang, Youngkeun Song

**Affiliations:** 1 Environmental Planning Institute, Seoul National University, Seoul 08826, Republic of Korea; 2 Graduate School of Environmental Studies, Seoul National University, Seoul 08826, Republic of Korea

**Keywords:** environmental science, environmental monitoring, environmental biotechnology, urban planning

## Abstract

Metabarcoding amplifies environmental DNA (eDNA) but often yields non-target taxa and false positives. Urban ecosystems are particularly prone to such detections due to inflows of external genetic material, yet their interpretation in urban eDNA studies remains unclear. This study examined non-target occurrences from fish-targeted metabarcoding in inland urban freshwater systems and proposes a perspective on interpreting such detections as ecologically meaningful signals rather than analytical noise. From 32 samples, 27.8% of total reads were non-targets, including 30 species from 21 families of non-target taxa and 35 species from 20 families of marine fish. Non-target taxa were most common in rivers, whereas marine fish appeared near wastewater facilities, reflecting human-derived inputs. These findings highlight that unexpected eDNA detections may reveal overlooked ecological and anthropogenic signals, offering insights for more reliable biodiversity assessment and management in urban ecosystems.

## Highlights

• Detected 30 non-target taxa and 35 marine species in urban freshwater systems
• Non-target signals revealed invasive species, waterbirds, and bats
• Marine fish detections were confined to sites near waste-processing facilities
• Seafood-related marine taxa indicate human activity signals in urban eDNA studies

## Graphical abstract

**Figure:**
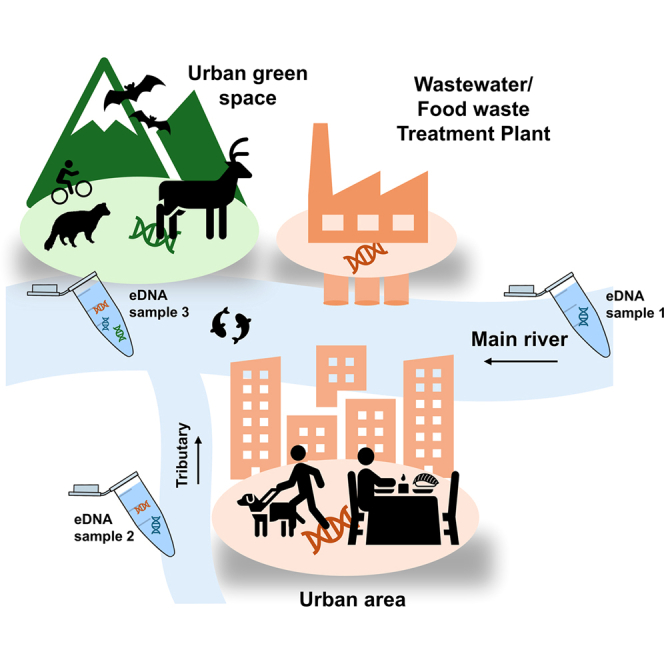

## Introduction

Environmental DNA (eDNA) metabarcoding is a high-throughput sequencing technique that enables the simultaneous detection of multiple organisms from a single environmental sample. It has been widely applied in studies of biological communities ranging from microorganisms to vertebrates. Based on species occurrence data, it has been used to assess biodiversity indices, evaluate ecological responses to environmental changes, and analyze ecological niches among species.^1^^,^^2^^,^^3^ However, because metabarcoding amplifies and analyzes genetic material from mixed environmental samples, it frequently contain signals that do not reflect the local target community.^4^^,^^5^ These unexpected detections arise from two conceptually distinct sources, which are often conflated in the literature.^6^ First, technical artifacts, generated by contamination, index/tag misassignment, or misidentification among closely related taxa, do not represent true biological presence and directly undermine data reliability^7^^,^^8^ (Table 1). Second, environmental signals, produced when DNA is transported from exogenous sources, represent biologically real but non-local signals.^9^ While non-target taxa are often excluded from analyses without major concern, false positives arising from sample contamination during field collection and laboratory processing, or from overestimation of closely related species during sequencing, directly affect data reliability and usability. Consequently, considerable research has focused on minimizing such errors in both field and laboratory environments.

**Table 1:** Conceptual and operational categories of unexpected eDNA detections in urban freshwater systems based on fish-targeted metabarcoding

Detection status	Category	Target taxa	Non-target taxa
Presence	Valid detection	Freshwater fish	Exogenous wildlife DNA (e.g., mammals, birds)
False presence	Technical artifacts	Misidentified or artifactual detections ∗ closely related species (e.g., congeners)	Same as target taxa
False presence	Environmental signals	Anthropogenic or transported DNA ∗ food waste, livestock, wastewater inputs	Same as target taxa

Urban ecosystems have recently been recognized as important spaces for biodiversity conservation. The designation of protected areas, expansion of green infrastructure, and restoration of aquatic ecosystems have improved ecological functions, connectivity, and biodiversity.^10^ Nevertheless, urban environments continue to face challenges such as non-point- and point-source pollution, anthropogenic development, and the increasing introduction and establishment of alien species.^11^^,^^12^^,^^13^ As complex spaces where multiple environmental factors and human activities overlap, urban ecosystems represent heterogeneous systems in which biodiversity and ecological functions can be interpreted from multiple perspectives. Accordingly, eDNA techniques have been increasingly applied to urban ecosystems.^14^^,^^15^ Unlike in natural ecosystems, urban environments often exhibit high frequencies of non-target detections due to the inflow of external genetic material. In cities where a wide range of organisms are raised and consumed, eDNA surveys frequently detect species not actually inhabiting the study site, and researchers often exclude such data during analysis. For example, Xiong et al.,^9^ reported that eDNA collected near urban wastewater treatment facilities contained DNA from ornamental species absent from the site. Similarly, Lee et al.,^16^ used eDNA from market areas to detect genetic diversity in commercially consumed fish species. On the other hand, several studies have utilized non-target data to identify alien or protected species, deriving additional ecological insights from information that is typically excluded.^7^ Thus, exogenous DNA introduced through human activities not only poses a risk of false-positive detections but also holds potential for monitoring urban environments and human impacts. To effectively remove or utilize such data, empirical studies evaluating the detection patterns of metabarcoding datasets in urban ecosystems are required.

Field sampling strategies are particularly important, as they can significantly influence eDNA results. Site selection depends on the target taxa, ecosystem characteristics, and research objectives. While many studies employ random sampling to ensure representativeness, researchers often exclude disturbed or atypical sites based on subjective judgment.^17^ Subsampling within a plot can capture fine-scale environmental variability, whereas pooling subsamples can increase sample representativeness. Hydrological flow is another critical factor: downstream sampling sites may yield more homogeneous detection results, yet upstream DNA does not necessarily accumulate downstream, underscoring the importance of prioritizing upstream sites.^18^ These considerations are closely linked to the detection of non-target taxa and the interpretation of reliable results. In this study, we evaluated the occurrence of non-target taxa from fish-focused metabarcoding results in an inland urban ecosystem and discussed strategies for field eDNA sampling and result interpretation in urban environments.

## Results

### Non-target detection patterns in urban ecosystems

**Figure 1:**
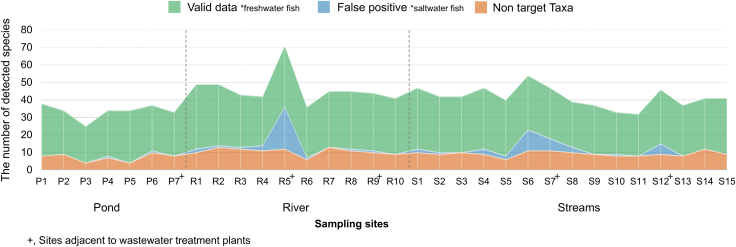
Stacked graph showing species detections according to detection type at each site

From 32 samples, a total of 10,371,838 reads were taxonomically assigned after quality filtering and sequence processing. Among these, 2,881,303 reads (27.8%) were identified as non-target taxa or potential false positives that could not be validated as freshwater fish (Tables S1 and S2). Non-target detections were classified into three categories: non-target taxa, freshwater false positives, and marine false positives, which accounted for 284,953 reads (2.7%), 2,578,566 reads (24.9%), and 17,784 reads (0.2%), respectively. In the negative control (DW), a total of 1,872 reads were generated; however, no sequences were taxonomically assigned, indicating the absence of biological amplification. Non-target detections were confirmed across all study sites (Figure 1). The mean number of non-target taxa detected per site was highest in rivers (10.7 ± 2.0), followed by streams (9.3 ± 1.4) and reservoirs (7.1 ± 2.2). In contrast, marine fish were concentrated on specific sites, including R5, S6, S7, and S12, where detection frequencies were particularly high.

**Figure 2:**
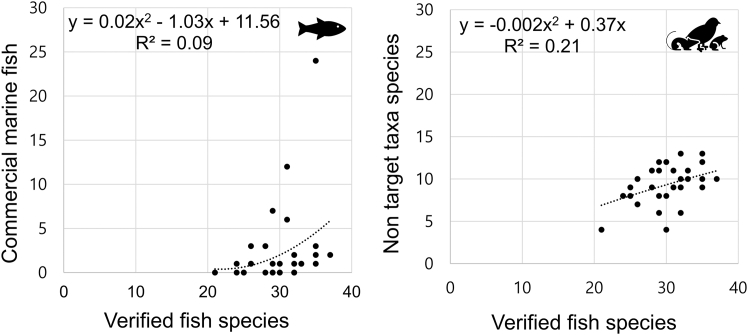
Correlation between valid data (freshwater fish), commercial marine fish, and non-target taxa based on polynomial regression analysis

Reservoirs, being isolated waterbodies, showed relatively low species richness, whereas rivers, which integrate inflows from multiple streams, yielded higher numbers of species. Sites with high frequencies of marine fish detections were located adjacent to wastewater treatment plants and food-waste treatment facilities, suggesting anthropogenic inputs as a likely source (Figure 1). As the number of validated species detections increased, there was a corresponding trend of higher detections of commercial marine fishes and non-target taxa. However, these relationships were not statistically significant (Figure 2). Thus, validated results remained robust for analysis regardless of changes in non-target detection frequency, alleviating concerns regarding potential species masking effects or the reduction of usable data due to non-target organisms.

### Species derived from non-target taxa and negative false-positive detection

**Figure 3:**
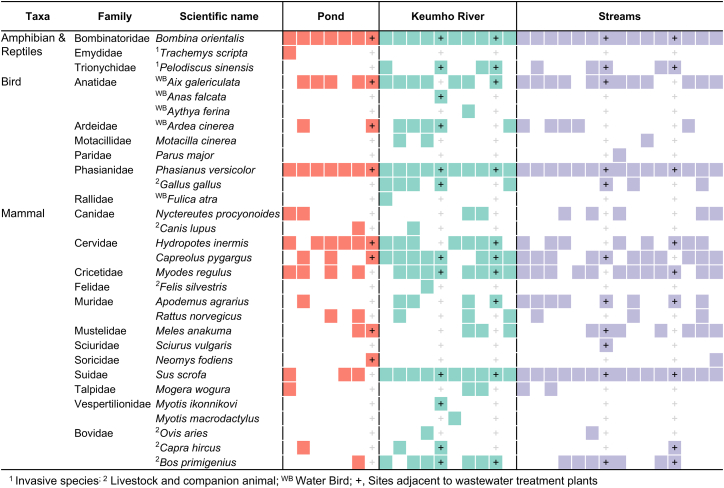
Species list of non-target taxa (non-fish species) according to sampling sites and environmental types

Analysis of non-target taxa revealed a total of 30 species from 21 families (Figure 3). These included three amphibian and reptile species, nine bird species, and 18 mammal species. Among amphibians and reptiles, the fire-bellied toad (*Lithobates catesbeianus*) was detected at all sites, while the red-eared slider (*Trachemys scripta*) and the Chinese softshell turtle (*Pelodiscus sinensis*) were detected at one pond site and nine river/stream sites, respectively. The red-eared slider is a well-known invasive alien species in Korea, introduced through ornamental use or religious release into rivers and lakes. Bird detections included five waterbird species and the gray wagtail (*Motacilla cinerea*), which typically breeds near wetlands,^19^ reflecting the habitat characteristics of both lotic and lentic environments. The common pheasant (*Phasianus colchicus*), a species with high urban densities, was detected across all sites, similar to the fire-bellied toad. Among mammals, two forest-dwelling bat species (*Myotis ikonnikovi* and *Myotis macrodactylus*) widely distributed in Korea were detected only at Geumho river sites located near forested areas (R5 and R6).^20^^,^^21^ Wild boar (*Sus scrofa*) showed higher detection frequencies in lotic environments. These results indicate the presence of diverse vertebrate groups within urban ecosystems and suggest that such detections may enhance the precision of urban biodiversity assessments. In addition, DNA from domestic animals such as dogs and cats, as well as livestock including cattle, goats, and sheep, was detected (Figure 3). These taxa are commonly raised in urban or peri-urban areas and consumed within the city, suggesting that their occurrence likely originated from non-point source inputs. The detections were consistent across site types, further supporting the influence of external anthropogenic sources.

**Figure 4:**
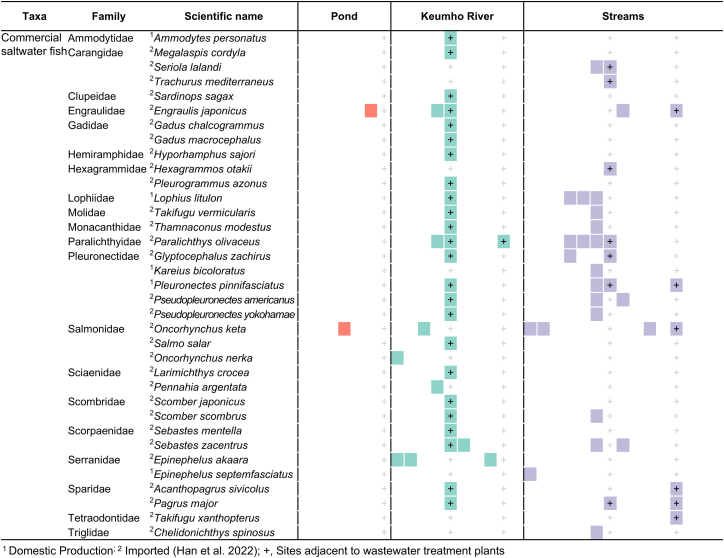
Species list of marine fish species according to sampling sites and environmental types

Among the detected fishes, approximately 12 freshwater species and 35 marine species (35 species from 20 families) were identified as potential false positives (Figure 4). Of these, 30 species were found to be imported into Korea, while five species are either naturally distributed or aquacultured in Korean waters. Notably, members of the families Scombridae (two species), Engraulidae (one species), Paralichthyidae (one species), Pleuronectidae (five species), and Salmonidae (three species) exhibited high DNA read abundances, corresponding to major seafood species consumed domestically (Ministry of Oceans and Fisheries 2023).

**Figure 5:**
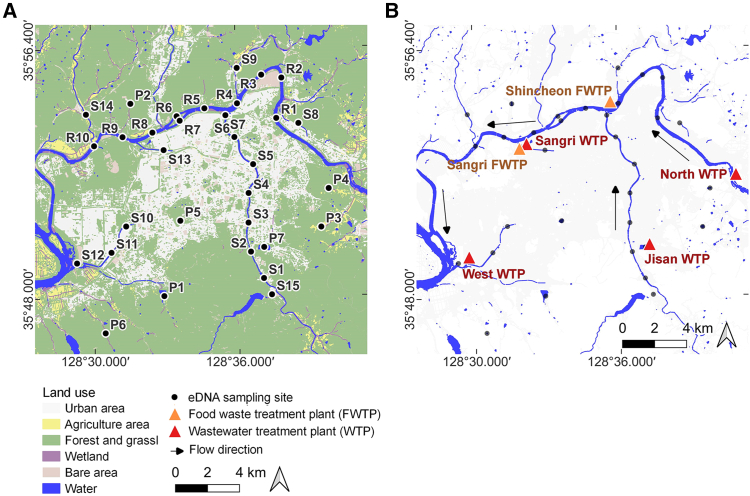
Overview of the study area, land-use characteristics, and eDNA sampling sites (A) Land-use map showing eDNA sampling sites (black circles) across major land-cover types within the hydrological system, including streams and reservoirs. (B) Locations of food-waste treatment plants (FWTPs; orange triangles) and wastewater treatment plants (WTPs; red triangles) in relation to the river network. Arrows indicate the flow direction of the river system.

Unlike livestock-associated DNA, the detection patterns of marine fishes were spatially concentrated, particularly at confluence sites (R5, S6–7, and S12). This suggests that food and waste treatment facilities located nearby may have introduced marine fish DNA at high concentrations into the waterbodies (Figures 1 and 5). These results demonstrate that in urban ecosystems, non-ecological factors such as human consumption activities can serve as major drivers of false-positive eDNA detections. At the same time, the findings highlight the need to establish filtering criteria and sampling strategies that explicitly account for the possibility of false positives in urban eDNA studies. Importantly, false-positive data should not be regarded merely as errors but also as potential sources of information that may provide insights into patterns of species consumption and human activities within urban environments.

## Discussion

A growing number of studies have explored the use of molecular bycatch—non-target taxa detected in metabarcoding—to obtain additional biological information. Macher et al.,^22^ reported that fish-targeted metabarcoding additionally detected 33 species of mammals and birds and suggested that repeated field sampling is necessary to increase such incidental detections. Similarly, Mariani et al.,^23^ demonstrated that bycatch data reflected surrounding landscapes and seasonality. In our study, non-target taxa were also frequently detected, and the patterns were consistent with local environmental conditions. For instance, waterbirds associated with wetlands and riparian zones were commonly detected, while forest-dwelling bats were detected only at river sites adjacent to forested areas (Figure 3). However, the MiFish primers used in this study target the 12 S region and are known to exhibit relatively limited amplification for non-fish vertebrates.^24^ Thus, primer-template mismatches may also contribute to detection bias and should be considered when interpreting non-target signals. Previous studies developing MiMammal and MiBird, which are modified derivatives of the MiFish primers, further highlight that primer modification can greatly enhance detection for particular taxonomic groups.^25^^,^^26^ Although MiFish has been successfully applied to monitor non-fish vertebrates in marine environments, effective assessment of specific taxonomic groups generally requires primers designed for the appropriate genetic markers.^27^^,^^28^ Nevertheless, such molecular bycatch can serve as a valuable complementary source of ecological information, reflecting surrounding habitat conditions in urban ecosystems and helping to identify areas that may warrant more targeted biological monitoring.

Our results also showed that false-positive errors are common in urban metabarcoding studies. Because the study area comprised inland freshwater ecosystems with well-defined habitat types, distinguishing false positives was relatively straightforward. However, taxa potentially introduced via livestock production and consumption were more difficult to evaluate as false positives. Moreover, in coastal cities or other landscape contexts, distinguishing false positives is expected to be even more challenging. Therefore, preventing or pre-filtering erroneous detections during site selection and data analysis is critical.

When selecting sampling sites, locations adjacent to waste-processing facilities, especially food waste treatment plants, should be avoided, or sampling should be conducted at least 3 km downstream of such facilities. This recommendation is supported by our observations that marine fish DNA was detected at high frequencies near food and wastewater treatment plants located at river confluences (e.g., S6–7 and R5), but detections dropped sharply at sites 2–3 km downstream (R6 and R7). However, the diffusion range of DNA depends on water velocity and discharge, and thus, in faster-flowing systems, sampling may need to occur at greater distances downstream.^29^^,^^30^ In addition, collecting subsamples near markets or waste-processing facilities, and selectively excluding taxa detected in those subsamples, may improve data reliability.

False positives often arise from results with extremely low read counts. Applying a threshold, such as excluding sequences representing less than 0.02% of the total amplification^31^ can effectively reduce such errors. In this study, the average read abundance of non-target taxa was 820.4 ± 11,047.4 reads, and for marine fish alone, 243.6 ± 385.4 reads. When applying a 0.02% threshold of total reads per sample, an average of 3.6 non-target taxa were removed, with up to 17 taxa excluded in some cases (Table S2).

When applying eDNA to urban ecosystem studies, data interpretation and analytical strategies should be carefully designed to align with research objectives and intended applications. Metabarcoding is well suited for capturing broad biodiversity patterns and characterizing taxonomic composition. However, to evaluate the ecological health of disturbed urban ecosystems, approaches beyond species richness alone are needed—specifically those that incorporate ecological groups or guilds.^32^ Sensitive and indicator species are particularly valuable, as they respond strongly to environmental changes and can serve as indirect measures of habitat quality and ecosystem health.^33^^,^^34^ Sensitive species react strongly to pollution, habitat fragmentation, and human activities, making their presence or detection frequency a critical indicator of ecological condition. Likewise, indicator species reflect specific environmental conditions such as water quality and substrate structure, and their distribution patterns can be used to quantitatively assess ecological stressors and human impacts in urban environments.^35^^,^^36^ For instance, the cyprinid fish Kumgang fat minnow (*Rhynchocypris kumgangensis*) is considered an indicator species sensitive to both water quality and climatic variation. It typically inhabits cold, fast-flowing streams with well-developed rocky and gravel substrates; consequently, its occurrence is constrained in urban waterways characterized by elevated temperatures and reduced flow velocity.^37^ Furthermore, the Eurasian otter (*Lutra lutra*), which favors river reaches with abundant prey resources and naturalized banks, functions as an umbrella species. Owing to its high mobility and trophic position, it serves as a reliable indicator for assessing ecological connectivity and overall ecosystem integrity in urban freshwater environments.^38^ As such, integrating sensitive and indicator taxa into targeted eDNA monitoring frameworks may enhance the ecological interpretability of urban assessments, complementing richness-based surveys with management-relevant information.

### Limitations of the study

This study analyzed non-target vertebrate detections by distinguishing signals that likely originated from resident wildlife from those introduced through environmental processes, focusing specifically on marine fishes as indicators of exogenous false-positive inputs. Freshwater species that are commercially utilized or potentially overestimated were excluded to minimize ambiguity; however, establishing a more systematic and quantitative framework to differentiate between true ecological presence and anthropogenic introduction remains necessary.

In addition, eDNA in lotic environments undergoes diffusion, transport, and sedimentation, yet hydrological factors were not explicitly considered in our analysis. External genetic materials discharged from wastewater treatment facilities may show variable detection probabilities depending on local hydrological conditions such as flow velocity, channel width, and water temperature. Future studies should incorporate hydrological modeling and DNA transport dynamics, as well as develop standardized criteria for distinguishing exogenous signals, to improve the interpretation of non-target eDNA detections in urban freshwater systems.

## Resource availability

### Lead contact

Requests for further information and resources should be directed to and will be fulfilled by the lead contact, Yujin Kang (yujin3384@gmail.com).

### Materials availability

This study did not generate new unique reagents.

### Data and code availability

• Raw sequence data from the metabarcoding dataset are available in the Sequence Read Archive (SRA: PRJNA1331475).
• No code was generated during this study.
• Any additional information required to reanalyze the data reported in this study is available from the lead contact upon request.

## Acknowledgments

This work was supported by the 10.13039/501100003725National Research Foundation of Korea (NRF) grant funded by the Korea government (10.13039/501100014188MSIT) (RS-2025-00562354).

## Author contributions

Conceptualization, Y.K. and Y.S.; methodology, Y.K.; investigation, Y.K.; formal analysis, Y.K.; writing – original draft, Y.K.; writing – review and editing, Y.K. and Y.S.; supervision, Y.S.; funding acquisition, Y.S.

## Declaration of interests

The authors have declared that no competing interests exist.

## Declaration of generative AI and AI-assisted technologies in the writing process

During the preparation of this work, the authors used ChatGPT5.1 in order to English language editing. After using this tool or service, the authors reviewed and edited the content as needed and take full responsibility for the content of the publication.

## STAR★Methods

### Key resources table

**Table:** 

REAGENT or RESOURCE	SOURCE	IDENTIFIER
**Biological samples**		

Water samples	This study	Table S3

**Critical commercial assays**		

DNeasy Blood & Tissue Kit	Qiagen	69504
MiSeq Reagent Kit v3 (600-cycle)	illumina	MS-102-3003
AMPure XP Beads	Beckman Coulter	A63880
Nextera XT Index Kit	illumina	FC-131-1002

**Deposited data**		

Raw sequence reads	This study	Sequence Read Archive (SRA) PRJNA1331475

**Oligonucleotides**		

MiFish-U primers F: 5′-GTCGGTAAAACTCGTGCCAGC -3′ R: 3′- GTTTGACCCTAATCTATGGGGTGATAC -5′	Miya et al.^39^	N/A

**Software and algorithms**		

MiFish pipeline (MitoFish)	Sato et al.^40^ and Iwasaki et al.^41^	https://mitofish.aori.u-tokyo.ac.jp/

### Experimental model and study participant details

Omitted, as this study did not involve experimental animals, human participants, cell lines, or laboratory-maintained biological models.

### Method details

#### Study sites and eDNA field sampling

This study aims to evaluate the potential occurrence of false positives across different types of aquatic environments within an urban setting (lotic vs. lentic). We focused on environmental characteristics of densely populated areas and adjacent treatment facilities to explore the influence of human activities and waste-processing operations that could generate false-positive signals.

The study area was Daegu Metropolitan City, a representative inland basin city in South Korea with a population of approximately 2.3 million. The Geumho river, the primary tributary of the Nakdong River, flows through the city, accompanied by 13 tributary streams. Sampling was conducted at a total of 32 sites, including 10 sites along the Geumho river mainstem (River, R1–R10), 14 sites in tributary streams (Stream, S1–S14; lotic environments), and 8 sites in lentic environments such as reservoirs and ponds within the urban area (Pond, P1–P8) (Figure 5A).

Within the sampling range, six waste-processing facilities were located, including food waste treatment plants (FWTPs) and wastewater treatment plants (WTPs) (Figure 5B). The Shincheon FWTP and WTP were located near site S7, West WTP near S12, Sangri FWTP and North WTP near S13, Ansim WTP near P4, and Jisan WTP near P7. These facilities are adjacent to major urban rivers and reservoirs, providing potential pathways for the inflow of exogenous DNA into aquatic systems.

For eDNA sampling, a total of 990 mL of water was filtered per site using three Sterivex filters (0.45 μm pore size, 330 mL per filter). This yielded a total of 96 filters across all sites. One negative control sample was included during the sampling process to monitor potential contamination. To prevent cross-contamination, independent 30 mL syringes and disposable gloves were used for each site. After filtration, Sterivex units were stored in an icebox to minimize DNA degradation and subsequently transported to the laboratory. Samples were stored at −20 °C until extraction. DNA extraction was performed following the protocol of the Qiagen DNeasy Blood & Tissue Kit (Qiagen, Hilden, Germany). Extracted DNA was quantified using a Nanodrop spectrophotometer (Nanodrop 2000c; Thermo Fisher Scientific, Waltham, MA, USA).

#### Library preparation and sequencing

To detect non-target taxa occurring in fish-targeted surveys, analyses were conducted using the MiFish universal primer set Miya et al.^39^ The metabarcoding protocol followed the guidelines of the eDNA Society manual.^42^ The NGS library was prepared using a two-step PCR with MiFish universal primers, followed by purification with Ampure XP beads and dual indexing using the Nextera system (Illumina). PCR conditions consisted of 30 cycles of denaturation at 98 °C, annealing at 50 °C, and extension at 68 °C. The indexed amplicons were quantified, normalized, and sequenced on the Illumina MiSeq platform using a v3 kit (2 × 300 bp).

#### Species identification and classification

Raw MiSeq reads were processed using the MiFish pipeline (http://mitofish.aori.u-tokyo.ac.jp/mifish). Forward and reverse reads were merged, primer sequences were trimmed, and low-quality or short reads (<140 bp) were discarded. After dereplication and chimera filtering, sequences were denoised and taxonomically assigned based on BLASTn searches against the MiFish database, using a 97% similarity threshold.

All organisms detected other than freshwater fish were classified as non-target taxa. Because the study sites were freshwater ecosystems located in inland cities, potential false-positive detections of freshwater fish were not evaluated. Such signals may originate from human consumption, occasional introductions, or taxonomic overestimation, making reliable interpretation difficult. Marine fish species were categorized according to whether they are consumed in Korea, based on Han et al.^43^ and the National Biodiversity Information System of Korea (https://species.nibr.go.kr/). Although taxonomic resolution could not be achieved at the subspecies level, several domesticated taxa were interpreted as domestic forms considering their likelihood of introduction in Korea and the characteristics of urban environments: *Canis lupus* (dog), *Bos primigenius* (cow), *Ovis aries* (sheep), and *Felis silvestris* (cat).

### Quantification and statistical analysis

Exploratory regression analyses were conducted in Microsoft Excel to evaluate the relationship between the number of validated species detections and the frequency of non-target detections, including commercial marine fishes. A polynomial regression was performed for descriptive purposes to examine overall trends in detection patterns. Sample size (n) represents the number of sampling sites. Regression results were used for qualitative interpretation.
