# Supplementary material for: Environmental insights from non-target detections in urban eDNA metabarcoding
Source: iScience. 2026 Jan 7;29(2):114632. doi: 10.1016/j.isci.2026.114632 (PMC12857387; doi:10.1016/j.isci.2026.114632)
Supplement: Document S1. Tables S1–S3 [file mmc1.pdf]

iScience, Volume *29*

Supplemental information

Environmental insights from non-target detections in urban eDNA metabarcoding

Yujin Kang and Youngkeun Song

Table S1. Validated species list of freshwater fish, related to Figure 1 and Figure 2
This table provides the list of freshwater fish species validated for downstream analyses, including scientific names and detection status across sites.

**Pond**								
Family	Scientific name	P1	P2	P3	P4	P5	P6	P7
Acheilognathinae	*Rhodeus uyekii*					8749		
	Rhodeus ocellatus ocellatus					16507		
Adrianichthyidae	*Oryzias latipes*	3039	2979	778	2108	2466	3558	3241
Amblycipitidae	*Liobagrus mediadiposalis*							
	Liobagrus andersoni	276	261		421	181	541	302
Centrarchidae	*Lepomis macrochirus*	8567	24446	86	71	62		48488
	Micropterus salmoides	13309	9390					3449
Channidae	*Channa argus kimurai*	687	678	487	770	181324	456	272
Cobitidae	*Misgurnus mizolepis*	282		51	1754	56	1452	92
	Misgurnus anguillicaudatus	724	1476	27	1082	889	917	617
	Koreocobitis rotundicaudata	1411	866	155	1149	1092	1066	287
	Paramisgurnus dabryanus	26176	2400	208	911	1154	4263	2478
	Iksookimia koreensis	227	193	36				
Cottidae	*Cottus poecilopus*	5261	2613	302	4217	2366	5571	3785
Cyprinidae	*Pseudopungtungia tenuicorpus*	891	882	57	1104	719	530	286
	Nipponocypris temminckii						7434	
	Rhynchocypris kumgangensis	63960	77217	7325	54953	46359	58832	67603
	Squalidus gracilis majimae	1853	1865	242	1794	1073	1395	2034
	Hemibarbus sp 090914		91			23	16	
	Pungtungia herzi				14			
	Gobiobotia brevibarba	463			91			140
	Carassius cuvieri	477	161		1501	429	6843	50
	Pseudogobio esocinus				183	189		
	Microphysogobio longidorsalis	406	507		276	270	339	292
	Rhynchocypris oxycephalus Korea	338		37	228	95	68	
	Coreoleuciscus splendidus	1334	1407	125	1414	910	1093	440

	Cyprinus carpio	3205	632			79		
	Pseudorasbora parva	38357	6982	85157	16279	14536	8550	22595
	Zacco platypus	41					108	
Gobiidae	*Tridentiger obscurus*							5609
	Rhinogobius sp BF	5744	25	42	176	7635	415	248
Nemacheilidae	*Lefua costata*	17661	24749	1881	14711	13392	15250	16827
	Barbatula toni	14270	22390	1709	16084	11379	16886	16759
Odontobutidae	*Odontobutis platycephala*	885						
	Odontobutis interrupta	97	740	54	125	7418	220	216
Osphronemidae	*Macropodus ocellatus*					93		
Salmonidae	*Oncorhynchus masou*	1361	4386	406	1372	1785	3241	2688
Siluridae	*Silurus microdorsalis*	698	346	20	622	178	711	472
Sinipercidae	*Coreoperca herzi*	212				637	2166	

River

Family	Scientific name	R1	R2	R3	R4	R5	R6	R7	R8	R9	R10
Acheilognathinae	*Acheilognathus macropterus*		62			281	902	161	3772	1692	2614
	Rhodeus ocellatus ocellatus										
Adrianichthyidae	*Oryzias latipes*	3702	3738	6734	2581	1443	546	1674	2618	2209	2584
Amblycipitidae	*Liobagrus andersoni*	752	206	382	583	313	84	101	236	155	39
Anguillidae	*Anguilla japonica*					1148					
Bagridae	*Tachysurus ussuriensis*									40	
Centrarchidae	*Lepomis macrochirus*	293	459	1162	361	221	96	248	366	865	1908
	Micropterus salmoides	704	1865	132	1621			488	442	241	2112
Channidae	*Channa argus kimurai*	643	806	638	723	264	94	1414	239	124	752
Cichlidae	*Oreochromis niloticus*										
Cobitidae	*Misgurnus mizolepis*	234	708		604	585	360	226		191	218
	Misgurnus anguillicaudatus	1401	863	2079	1755	577	25	270	1352	497	328
	Koreocobitis rotundicaudata	690	1467	864	1836	156	58	230	951	580	1198
	Koreocobitis naktongensis	593	819		148	69			122	26	43

	Paramisgurnus dabryanus	1772	1874	1697	3018	12346	501	595	2017	2270	3515
	Iksookimia koreensis	87	90				17			60	
Cottidae	*Cottus poecilopus*	4413	4102	3160	4534	1105	483	1125	3111	2794	2993
Cyprinidae	*Pseudopungtungia tenuicorpus*	683	898	987	1439	504	43	333	1039	431	816
	Nipponocypris temminckii					54	51				
	Rhynchocypris kumgangensis	54967	47600	52328	93069	35844	10165	23020	64882	33931	60803
	Squalidus gracilis majimae	1165	1373	1909	1838	917	165	386	1446	568	645
	Opsariichthys uncirostris uncirostris	82	117	89	739	77		930	525	252	
	Hemibarbus sp 090914	9858	244			671					814
	Pungtungia herzi	607	298			1620	11	99	195	18	
	Microphysogobio yaluensis	267	1650	56		173		99	373		688
	Gobiobotia brevibarba	111		186							87
	Carassius cuvieri	358		4536	730	70					
	Pseudogobio esocinus	284	971	132	59	1136	60	2109	3070	1282	2177
	Squalidus japonicus coreanus	5945	2127	942	1988	147	59	1413	1026	2296	3733
	Squalidus sp SX-0527		153	142			39		48		
	Microphysogobio longidorsalis	173		257	399			74	75	49	
	Rhynchocypris oxycephalus Korea	42	128	36			16		124	82	
	Coreoleuciscus splendidus	1664	1199	995	2296	745	152	261	1153	1138	1075
	Cyprinus carpio	312				542		7379		9228	3129
	Pseudorasbora parva	23957	176210	27564	10768	49234	538209	203836	59984	159936	75059
	Zacco platypus	673	5322	3282	152	3577		500	1548	829	1961
Gobiidae	*Rhinogobius giurinus*						35				
	Tridentiger obscurus							48			411
	Rhinogobius sp BF	6124	2638	137	4668	8295	313	2578	22211	3653	20025
Nemacheilidae	*Lefua costata*	14677	12114	15812	27356	10443	2832	5516	18079	7718	14372
	Barbatula toni	14584	11306	12520	18554	8324	2199	4725	12555	9558	11074
Odontobutidae	*Odontobutis platycephala*	147				508		80	838		349
	Odontobutis interrupta	664	84750	268	414	11219	221	2613	4846	18250	7069
Salmonidae	*Oncorhynchus masou*	2827	2334	1771	5545	839	424	1246	2023	1577	1640

Siluridae	*Silurus microdorsalis*	356	140	272	511		38	171	685	317	318					
Sinipercidae	*Coreoperca herzi*	6918	106			71			247							
	Siniperca scherzeri		227													

Stream

Family	Scientific name	S1	S2	S3	S4	S5	S6	S7	S8	S9	S10	S11	S12	S13	S14	S15
Acheilognathinae	*Acheilognathus chankaensis*	95														
	Acheilognathus macropterus	50														
Adrianichthyidae	*Oryzias latipes*	4068	1891	6961	1691	1390	4134	5801	1977	4634	8190	4271	1814	2186	1203	4159
Amblycipitidae	*Liobagrus mediadiposalis*		11	21												
	Liobagrus andersoni	60	479	318	54	220	521			700	431	49	32	368	170	362
Anguillidae	*Anguilla japonica*				81				30							
Bagridae	*Tachysurus fulvidraco*													44	16	
Centrarchidae	*Lepomis macrochirus*	181	162	275		63		145	655			163	2167	209	8411	206
	Micropterus salmoides	25							1419				496	3265	3197	
Channidae	*Channa argus kimurai*	399		503	143	234	279	375	1009	342	310	460	22386	531	1064	618
Cichlidae	*Oreochromis niloticus*												116			
Cobitidae	*Misgurnus mizolepis*	4709	3842	2754	1297	1854	2412	1396	1668	16620	36895	100614	14464	2662	11	1602
	Misgurnus anguillicaudatus	2918	1683	1174	483	703	426	1910	13440	2396	1230	2926	472	1379	880	1355
	Koreocobitis rotundicaudata	542	769	1739	588	373	2335	1382	516	2127	1394	559	447	189	589	1039
	Kichulchoia multifasciata	10273	636	698	634	104										55
	Paramisgurnus dabryanus	3084	4097	3261	1423	1600	3285	3771	306	2597	1964	1017	1579	1902	880	3426
	Iksookimia koreensis					48	150		64	264		128	135			
Cottidae	*Cottus poecilopus*	3513	3673	4961	1429	2161	1616	3784	1618	3114	4044	3596	2697	4224	2067	6155
Cyprinidae	*Pseudopungtungia tenuicorpus*	803	1138	1360	572	584	1274	1581	261	441	1200	139	623	641	559	1668
	Nipponocypris temminckii	21710	3045	2611	1320	1369	224	188								1763
	Rhynchocypris kumgangensis	64478	69119	71904	31165	30448	47886	59421	22664	64734	66862	55403	41933	47213	32217	93468
	Squalidus gracilis majimae	1883	2334	1915	666	751	1410	1007	792	1960	1663	1183	1039	875	1020	3539
	Opsariichthys uncirostris uncirostris														1091	
	Hemibarbus sp 090914		83		149					32			154			34

	Pungtungia herzi	58138	51487	17893	41756	77043	31826	32801			182					6249
	Microphysogobio yaluensis													398		
	Gobiobotia brevibarba				59	24	276		74		373	170		128	126	99
	Carassius cuvieri	42	382		165					5149	461	629				90
	Pseudogobio esocinus		89	264	24	211	893	1914					176	1761	1404	
	Squalidus japonicus coreanus	6191	11024	11201	5128	4283	3604	2388		15440			288	41	443	
	Microphysogobio longidorsalis	209	322		129	26	375	205	171	385	389	169	192	18		486
	Rhynchocypris oxycephalus Korea		217		83	48	101	144	36045	383	368		368		128	24
	Carassius auratus langsdorfii									93						
	Carassius sp	259														502
	Coreoleuciscus splendidus	1635	2007	1827	495	582	1458	1259	305	675	764	979	784	777	944	1371
	Cyprinus carpio	640		12631	8756	5003	3555	3008				97	23808	899	24432	
	Pseudorasbora parva	5384	1223	6503	16572	42217	87928	114638	235763	11648	946	2639	166322	279694	95048	1692
	Zacco platypus	7158	1395	1804	4918	2721	15919	6145	6015	10362			349	2540	650	1091
Gobiidae	*Rhinogobius giurinus*			31												
	Tridentiger obscurus	65		75	98								7468			
	Rhinogobius sp BF	264	154	1339	770	1059	2048	5193		2617	333	115	2806	11403	959	157
Nemacheilidae	*Lefua costata*	18924	19063	18775	8686	9818	12473	15383	21373	18882	14175	12724	11015	13817	9561	30800
	Barbatula toni	15747	15010	18580	7153	7475	13121	14755	6152	13084	17022	11049	8393	12861	7612	21110
Odontobutidae	*Odontobutis platycephala*	22446	45761	2340	875	995	952	1180		3339						12044
	Odontobutis interrupta		47	15	159		50	474	2695	56676	128		23120	1063	990	688
Osphronemidae	*Macropodus ocellatus*								26							
Salmonidae	*Oncorhynchus masou*	895	3859	3175	1233	2202	2060	2017	942	3668	3511	2136	1607	1493	902	2072
Siluridae	*Silurus microdorsalis*	626	309	621	583	231	341	367	425	317	564	679	315	392	64	314
Sinipercidae	*Coreoperca herzi*	6229	8574	447	278	215	103	115								

Table S2. Non-target vertebrate species list, related to Figure 1 and Figure 2
This table summarizes all non-target vertebrate taxa detected in the metabarcoding dataset, including birds, mammals, reptiles, and amphibians. The table includes scientific names and detection status across sites.

Pond									
**Taxa**	**Family**	**Scientific name**	**P1**	**P2**	**P3**	**P4**	**P5**	**P6**	**P7**
Amphibians and Reptiles	Bombinatoridae	*Bombina orientalis*	442	1543	161	1534	949	1220	1065
	Emydidae	*Trachemys scripta*	148						
Birds	Anatidae	*Aix galericulata*		109	22	17		252	260
	Ardeidae	*Ardea cinerea*		10					82
	Phasianidae	*Phasianus versicolor*	556	511	13	559	329	219	83
Mammals	Canidae	*Nyctereutes procyonoides*	49	403					
		Canis lupus						52	
	Cervidae	*Hydropotes inermis*	233		42	28	170	222	105
		Capreolus pygargus		95		82			42
	Cricetidae	*Myodes regulus*	81	93		148		450	
	Muridae	*Apodemus agrarius*		58					
		Rattus norvegicus				55		136	
	Mustelidae	*Meles anakuma*						48	66
	Soricidae	*Neomys fodiens*							49
	Suidae	*Sus scrofa*	134				355	729	
	Talpidae	*Mogera wogura*	214						
	Bovidae	*Capra hircus*		73					
		Bos primigenius						276	
Marine Fish	Engraulidae	*Engraulis japonicus*						100	
	Salmonidae	*Oncorhynchus keta*				338			
False positive freshwater fish	Cyprinidae	*Hemibarbus umbrifer*	343	202					
		Carassius gibelio	48482	26758	525109	61345	29558	103981	9879
		Cyprinus megalophthalmus					80	106	
		Culter dabryi							384
		Tribolodon sachalinensis			17			76	204

	Siluridae	*Silurus soldatovi*	110									
	Salmonidae	*Brachymystax savinovi*	8193	12236	1008	8830	6793	7833	5064			

River

**Taxa**	**Family**	**Scientific name**	**R1**	**R2**	**R3**	**R4**	**R5**	**R6**	**R7**	**R8**	**R9**	**R10**
Amphibians and Reptiles	Bombinatoridae	*Bombina orientalis*	991	1177	644	1450	461	221	528	1843	846	892
	Trionychidae	*Pelodiscus sinensis*	59				69			9	43	
Birds	Anatidae	*Aix galericulata*	196	159	236	69		51	69		125	
		Anas falcata					31					
		Aythya ferina							15			
	Ardeidae	*Ardea cinerea*		11	108	136	32					10
	Motacillidae	*Motacilla cinerea*		66		35						
	Phasianidae	*Phasianus versicolor*	208	471	117	231	146	40	91	357	133	54
		Gallus gallus	112	86	38		18					80
	Rallidae	*Fulica atra*	49									
Mammals	Canidae	*Nyctereutes procyonoides*							159	14		
		Canis lupus			38							
	Cervidae	*Hydropotes inermis*	170	74	130			40	22	272	135	
		Capreolus pygargus	116	91	242	195	37			76	13	28
	Cricetidae	*Myodes regulus*		270	168	351	126		89	128	224	54
	Felidae	*Felis silvestris*				28						
	Muridae	*Apodemus agrarius*		154	95				37		268	
		Rattus norvegicus		65					30			83
	Mustelidae	*Meles anakuma*							49	140		59
	Suidae	*Sus scrofa*	292	10	296	311	212640	76	403	2092	489	248
	Talpidae	*Mogera wogura*							16	34		
	Vespertilionidae	*Myotis ikonnikovi*					47					
		Myotis macrodactylus						33				
	Bovidae	*Ovis aries*				123						
		Capra hircus		68			26					

		Bos primigenius	254		57	40	292		11	18	86
Marine Fish	Ammodytidae	*Ammodytes personatus*					74				
	Carangidae	*Megalaspis cordyla*					67				
	Clupeidae	*Sardinops sagax*					62				
	Engraulidae	*Engraulis japonicus*				13	2111				
	Gadidae	*Gadus chalcogrammus*					101				
		Gadus macrocephalus					731				
	Hemiramphidae	*Hyporhamphus sajori*					51				
	Hexagrammidae	*Pleurogrammus azonus*					38				
	Lophiidae	*Lophius litulon*					199				
	Molidae	*Takifugu vermicularis*					50				
	Monacanthidae	*Thamnaconus modestus*					67				
	Paralichthyidae	*Paralichthys olivaceus*				317	215				79
	Pleuronectidae	*Glyptocephalus zachirus*					20				
		Pleuronectes pinnifasciatus					477				
		Pseudopleuronectes americanus					61				
		Pseudopleuronectes yokohamae					192				
	Salmonidae	*Oncorhynchus keta*			33						
		Salmo salar					216				
		Oncorhynchus nerka	245								
	Sciaenidae	*Larimichthys crocea*					22				
		Pennahia argentata				81					
	Scombridae	*Scomber japonicus*					104				
		Scomber scombrus					1924				
	Scorpaenidae	*Sebastes mentella*					92				
		Sebastes zacentrus					215	34			
	Serranidae	*Epinephelus akaara*	755	24						170	
	Sparidae	*Acanthopagrus sivicolus*					215				

		Pagrus major					216					
False positive freshwater fish	Cyprinidae	*Hemibarbus umbrifer*	26816	13305	11155	8348	17414	175	6244	19970	2941	4760
		Hemibarbus maculatus							69			
		Plagiognathops microlepis			4092	358	45	94	30		2846	1126
		Carassius gibelio	9647	37521	136838	16157	17699	16076	180448	15351	49379	13526
		Culter dabryi	663	190	456	595	110	64	661	3725	374	320
		Tribolodon sachalinensis	183		217		114			313	71	18
	Siluridae	*Silurus meridionalis*					24					
		Silurus soldatovi		110			101		25	180	26	37
	Cobitidae	*Misgurnus bipartitus*				911	87				36	51
	Leuciscidae	*Rhynchocypris percnurus sachalinensis*							51			
	Salmonidae	*Brachymystax savinovi*	9109	5556	6056	8539	3414	958	3428	7212	4111	6559

Stream

Taxa	Family	Scientific name	S1	S2	S3	S4	S5	S6	S7	S8	S9	S10	S11	S12	S13	S14	S15
Amphibians and Reptiles	Bombinatoridae	*Bombina orientalis*	1295	1636	1086	582	270	752	1270	445	798	1078	1168	828	1358	1097	920
	Trionychidae	*Pelodiscus sinensis*		22				62	49				32	90			
	Anatidae	*Aix galericulata*	70	99	80	163		239	556	38	211	22			111	143	117
Birds	Ardeidae	*Ardea cinerea*	131		55	44	33								23		
	Motacillidae	*Motacilla cinerea*										32					
	Paridae	*Parus major*								58							
	Phasianidae	*Phasianus versicolor*	111	176	807	185	166	186	30	24	959	257	255	202	183	138	179
		Gallus gallus							59		81					55	
Mammals	Canidae	*Nyctereutes procyonoides*				54		200		110						55	84
	Cervidae	*Hydropotes inermis*	375		177	29				24		94		195	47	18	
		Capreolus pygargus	107	294	49			311	162		453	356	166		137	27	147
	Cricetidae	*Myodes regulus*	149	176	193		154	38		172	259	87	19	248		114	145
	Muridae	*Apodemus agrarius*	178	285	297	102			186		75			42		323	
		Rattus norvegicus		209							66					69	
	Mustelidae	*Meles anakuma*						102	210	79			139		37	122	94

	Sciuridae	*Sciurus vulgaris*							228								
		Neomys fodiens															232
	Suidae	*Sus scrofa*	8	533	947	66	170	964	353	43	322	14	13	269	196	539	253
	Talpidae	*Mogera wogura*	170		54												
	Bovidae	*Ovis aries*						9									
		Capra hircus												61			
		Bos primigenius				185	79	285	18	36			17	116			
Marine Fish	Carangidae	*Seriola lalandi*						334	244								
		Trachurus mediterraneus							40								
	Engraulidae	*Engraulis japonicus*								105				60			
	Hexagrammidae	*Hexagrammos otakii*							140								
	Lophiidae	*Lophius litulon*				13	15	194									
	Molidae	*Takifugu vermicularis*						235									
	Monacanthidae	*Thamnaconus modestus*						76									
	Paralichthyidae	*Paralichthys olivaceus*				81	69	375	405								
	Pleuronectidae	*Glyptocephalus zachirus*				24			61								
		Kareius bicoloratus						576									
		Pleuronectes pinnifasciatus						39	275					66			
		Pseudopleuronectes americanus						1349		12							
		Pseudopleuronectes yokohamae						1189									
	Salmonidae	*Oncorhynchus keta*	70	200								138		151			
	Scombridae	*Scomber scombrus*						145									
	Scorpaenidae	*Sebastes zacentrus*						8		35							
	Serranidae	*Epinephelus septemfasciatus*	66														
	Sparidae	*Acanthopagrus sivicolus*												126			
	Sparidae	*Pagrus major*							298					59			
	Tetraodontidae	*Takifugu xanthopterus*												106			
	Triglidae	*Chelidonichthys spinosus*						696									

False positive freshwater fish	Cichlidae	*Oreochromis macrochir*													30		
	Cyprinidae	*Hemibarbus umbrifer*			12	204	34		1376	190	52			5555	263	533	
		Plagiognathops microlepis												428	621	60	
		Carassius gibelio	11127	6547	8435	113035	147225	37818	68145	207201	14187	7683	33008	10189	5947	238978	12153
		Cyprinus megalophthalmus		292								196					
		Culter dabryi			764									221			
		Tribolodon sachalinensis		363			57					737	89		217	137	290
	Siluridae	*Silurus meridionalis*															
		Silurus soldatovi	141	87	37	94	16	100	67	11	393			54	785		481
	Cobitidae	*Misgurnus bipartitus*	485		118	103	123	734			357	119	2411	105			310
	Salmonidae	*Brachymystax savinovi*	6794	7576	9695	4552	3569	6004	7345	4173	5530	8219	7640	6436	8389	3695	13743

Table S3. Sampling sites and water quality, related to Figure 5
This table summarizes the geographic information of each sampling site and associated water quality parameters, including temperature, dissolved oxygen, conductivity, salinity and pH.

Site	X	Y	Temp (℃)	DO (mg/L)	Cond (μS/cm)	Sal	pH
P1	128.54836	35.80080	16.8	8.7	160.7	0.1	8.6
P2	128.52694	35.90926	_	_	_	_	_
P3	128.65759	35.83850	17.1	7.7	506.1	0.3	8.4
P4	128.66315	35.86012	14.9	8.9	273.4	0.1	9.3
P5	128.56022	35.84304	16.0	7.8	286.3	0.1	8.4
P6	128.50745	35.78035	17.0	6.0	180.2	0.1	8.3
P7	128.61791	35.82760	16.0	7.9	335.7	0.2	8.4
R1	128.62767	35.90004	15.3	11.1	651.3	0.3	8.1
R10	128.50138	35.88571	23.2	18.6	2473.3	1.3	7.8
R2	128.63166	35.92269	14.6	12.6	661.6	0.3	8.0
R3	128.61777	35.92449	16.5	11.3	646.8	0.3	7.8
R4	128.60070	35.90860	14.8	10.8	651.3	0.3	8.0
R5	128.57808	35.90607	16.7	12.0	505.8	0.3	8.0
R6	128.55878	35.90185	19.4	12.7	579.0	0.3	8.6
R7	128.56085	35.89931	18.6	13.3	610.5	0.3	8.6
R8	128.54187	35.89286	16.5	10.7	588.1	0.3	8.4
R9	128.52116	35.89054	18.1	11.8	616.0	0.3	8.2
S1	128.61743	35.81010	16.1	8.5	223.4	0.1	8.2
S2	128.60874	35.82505	11.1	8.8	217.3	0.1	8.5
S3	128.60734	35.84142	13.0	9.5	355.5	0.2	8.2
S4	128.60772	35.85806	14.4	7.6	467.7	0.2	8.0
S5	128.61117	35.87424	13.7	9.9	453.2	0.2	8.0
S6	128.59852	35.88968	13.0	10.5	467.9	0.2	8.0
S7	128.59253	35.90202	13.7	8.9	501.1	0.2	7.9
S8	128.64294	35.89701	17.2	6.6	577.3	0.3	8.0
S9	128.60088	35.92853	15.2	9.6	634.7	0.3	7.9

S10	128.52273	35.84028	26.5	15.4	609.5	0.3	9.1
S11	128.51232	35.82563	25.1	33.8	467.8	0.2	9.5
S12	128.48840	35.81987	20.5	6.6	911.0	0.4	8.3
S13	128.54942	35.88292	23.2	18.4	2803.1	1.5	7.8
S14	128.49608	35.90349	16.5	10.7	488.1	0.3	8.4
S15	128.62277	35.80086	14.0	7.4	176.9	0.1	8.1
